# Supplementary material for: Reshaping reservoirs with unsupervised Hebbian adaptation
Source: Nat Commun. 2025 Dec 13;17:450. doi: 10.1038/s41467-025-67137-1 (PMC12800270; doi:10.1038/s41467-025-67137-1)
Supplement: Supplementary file 1 — Supplementary Information file [file 41467_2025_67137_MOESM1_ESM.pdf]

Reshaping reservoirs with unsupervised Hebbian adaptation : Supplementary Information

Tanguy Cazalets and Joni Dambre

*Corresponding author(s). E-mail(s): tanguy.cazalets@ugent.be;

Contents

A Details on HAG Algorithm **2**
- A.1 Pseudo-code for the HAG algorithm 2
- A.2 Linear Correlation Coefficient 3
- A.3 Differential Realization of Signed Weights for Positive-Only Reservoirs 3

B Details on the hyperparameter optimization **5**
- B.1 Hyperparameter Definitions and range: 5
- B.2 Hyperparameter Optimization Framework 7
- B.3 Optimized Hyperparameters for Each Algorithm 7
- B.4 Cross validation performances 11

C Additional Analysis **12**
- C.1 On Memory Capacity in a Multivariate Setting 12
- C.2 Computational Complexity 12
- C.3 Estimation of computation costs 15
- C.4 Final connectivity details 16

D Sensitivity to the Modularization of the Input Mapping **18**

E Detailed Analysis Results **20**

Appendix A Details on HAG Algorithm

A.1 Pseudo-code for the HAG algorithm

Algorithm 1 HAG Algorithm

Require: Reservoir weights $\mathbf{W}$, Input weights $\mathbf{W}_{\text{in}}$, Bias $\mathbf{b}$, Pretraining data X_{pretrain}, Hyperparameters $(\rho, \beta, \delta w, \gamma, T_{\min}, T_{\max})$
Ensure: Adjusted reservoir weights $\mathbf{W}$

while (STREAMING and remaining $> T_{\max}$) **or** (FULLINSTANCE and instances remain) **do**
  if FULLINSTANCE **then**
    $T_{\text{current}} \leftarrow$ length of next instance
  else
    Draw T_{current} uniformly from logspace($T_{\min}$, $T_{\max}$, 10)
  end if
  for $t \leftarrow 1$ to T_{current} **do**
    Update reservoir states:
    $\mathbf{x}[t+1] \leftarrow \sigma\left(\mathbf{W}\mathbf{x}[t] + \mathbf{W}_{\text{in}}\mathbf{u}[t] + \mathbf{b}\right)$
  end for
  for each neuron i **do**
    Compute activity measure s_i (mean or variance over T_{current})
    Compute growth indicator:
    $\Delta z_i \leftarrow \frac{1}{\beta}\left(s_i - \rho\right)$
    if $\Delta z_i < -1$ **then**
      Find neuron j with highest linear correlation with neuron i
      Increase weight:
      $w_{ij} \leftarrow w_{ij} + \delta w$
    end if
    if $\Delta z_i > +1$ **then**
      Randomly select a synapse w_{ij} connected to neuron i to decrease
      $w_{ij} \leftarrow \max(w_{ij} - \delta w, 0)$ ▷ Ensure non-negativity
    end if
    if variance-HAG and x_i exceeds saturation threshold θ_{sat} **then**
      for each outgoing synapse w_{ij} of neuron i **do**
        $w_{ij} \leftarrow w_{ij} \times \eta_{\text{sat}}$
      end for
    end if
  end for

A.2 Linear Correlation Coefficient

To dynamically form connections in the reservoir, we identify neurons that exhibit strong linear relationships (Pearson, 1895) in their activation patterns. This process is performed exclusively on neurons that have not yet achieved homeostasis, as defined by the growth indicator Δz.

For two neurons i and j, the linear correlation coefficient, r_{ij}, measuring the linear relationship between their respective activation states, $x_i[t]$ and $x_j[t]$, over a time period T is defined as:

$$r_{ij} = \frac{\sum_{t=1}^{T}(x_i[t] - \bar{x}_i)(x_j[t] - \bar{x}_j)}{\sqrt{\sum_{t=1}^{T}(x_i[t] - \bar{x}_i)^2}\sqrt{\sum_{t=1}^{T}(x_j[t] - \bar{x}_j)^2}}, \tag{A1}$$

where:

- $x_i[t]$ and $x_j[t]$ are the activation states of neurons i and j at time t,
- $\bar{x}_i = \frac{1}{T}\sum_{t=1}^{T} x_i[t]$ is the mean activation state of neuron i over the period T,
- $\bar{x}_j = \frac{1}{T}\sum_{t=1}^{T} x_j[t]$ is the mean activation state of neuron j over the same period.

The coefficient r_{ij} ranges from -1 (perfect negative correlation) to 1 (perfect positive correlation), with 0 indicating no linear relationship.

A.3 Differential Realization of Signed Weights for Positive-Only Reservoirs

Many physical substrates naturally provide only non-negative signals or couplings (optical intensities, conductances). To implement signed linear combinations on such hardware, a common approach is to decompose each weight into a difference of two non-negative parts:

$$w = w^+ - w^-, \qquad w^+ \geq 0,\ w^- \geq 0,$$

so that for a reservoir state $\mathbf{x} \in \mathbb{R}^n$ the readout computes

$$y = \sum_{i=1}^{n} w_i x_i = \sum_i w_i^+ x_i \ - \ \sum_i w_i^- x_i,$$

yielding an *effective* signed weight using only positive-valued components.

Platforms and realizations.

- **Photonic (intensity).** Each feature is split into two optical paths, with separate weights on each. A balanced photodetector subtracts the photocurrents, producing effective positive and negative contributions while canceling common-mode noise (Wang et al, 2024; Abreu et al, 2024).
- **Memristive / resistive crossbars.** A synaptic weight is represented by two conductances G^+ and G^- on separate columns. The difference of the resulting currents, $I \propto G^+ - G^-$, realizes an effective signed weight using only positive-valued conductances (Kudithipudi et al, 2016).

Trade-offs.

Differential encoding requires two physical paths per weight (doubling area or device count), careful gain/phase matching, and reduces dynamic range per branch. In return, it enables signed operations using positive-only primitives and improves signal-to-noise via common-mode cancellation—a favorable trade-off for HAG's excitatory reservoirs.

Implications for HAG.

HAG constrains the reservoir's recurrent matrix W to be non-negative ($W \geq 0$), making it compatible with hardware that cannot natively implement negative couplings. Signed computation is delegated to the linear readout (and, if needed, between-module couplings) by using differential encoding, preserving the readout's expressivity while keeping the reservoir hardware-friendly.

Appendix B Details on the hyperparameter optimization

This appendix details the optimized hyperparameters across datasets to support reproducibility and highlight how parameter choices affect algorithm performance.

B.1 Hyperparameter Definitions and range:

Table B1: Hyperparameter ranges, settings, and notation.

CATEGORY	PARAMETER	SYMBOL	RANGE/SETTINGS
**Fixed Parameters**	Activation Function	σ	Hyperbolic Tangent (tanh)
	Input Connectivity	-	1 (Fully Connected)
	Network Size	n	Just above 500 neurons
	Input Duplication	-	Equal duplication per input
**Shared**	Input Scaling	s_{in}	0.01 - 0.2, step 0.005
	Bias Scaling	s_b	0 - 0.2, step 0.005
	Ridge Coefficient	λ	10^{-15} - 10^{1}, log scale
**E-ESN/ESN**	Connectivity	p	0 - 1
	Spectral Radius	ρ_s	0.4 - 1.6, step 0.01
**IP**	Target Distribution Mean	μ	0 – 1
	Target Distribution Variance	σ_{ip}	0 – 1
	Learning rate IP	η_{ip}	10^{-6} – 10^{-1} (log)
**Anti-Oja**	Learning rate Anti-Oja	η_{oja}	10^{-5} – 10^{-1} (log)
**Shared-HAG**	Maximum Degree	γ	None
	Weight Increment	δw	0.001 - 0.1, step 0.001
	Adaptive Time Window	T	Data-driven (see below)
**mean-HAG**	Rate Target	ρ_r	0.5 - 1, step 0.01
	Rate Spread	β_r	0.01 - 0.4, step 0.005
**variance-HAG**	Variance target	ρ_v	0.001 – 0.020 (step 0.001)
	Variance spread	β_v	0.001 – 0.020 (step 0.001)
	Saturation threshold	θ_{sat}	0.80 – 0.98 (step 0.02)
	Scaling after saturation	η_{sat}	0.80 – 0.98 (step 0.02)
**LSTM/GRU**	Hidden size	n	128 – 512 (step 32)
	# Recurrent layers	N_{layers}	1 (fixed)
	Dropout	p_{drop}	0.0 – 0.5
	Bidirectional flag	b	$\{\text{False}, \text{True}\}$
	Learning rate (Adam)	η	10^{-10} – 10^{-1} (log)
	Batch size	B	$\{8, 16, 32, 64, 128, 256, 512\}$
	Training epochs	E	5 – 20

A. ESN-wide hyper-parameters

- **Input scaling** (s_{in}): Scaling factor applied to the input weights W_{in}.
- **Bias scaling** (s_b): Scaling factor applied to the bias vector $\mathbf{b}$.

- **Ridge coefficient** (λ): Regularization parameter in ridge regression, where $\lambda = 10^{\text{ridge exponent}}$.

B. Adaptation-specific hyper-parameters

(only active for the indicated reservoir type)

- *Intrinsic Plasticity (IP):*
  - **Mean of Target Distribution for IP (μ):** Target mean for intrinsic plasticity normalization.
  - **Variance of Target Distribution for IP ($\sigma_{\mathbf{ip}}$):** Target standard deviation for intrinsic plasticity normalization.
  - **Learning rate** (η_{ip}).
- *Anti-Oja:* **Learning rate** (η_{oja}).
- *HAG (shared):*
  - **Weight increment** (δw) – amount by which synaptic weights are increased or decreased during adaptation.
  - **Maximum degree** (γ) – optional cap on number of synaptic partners per neuron.
  - **Adaptive time window** ($[T_{\min}, T_{\max}]$) – At each adaptation step we either (i) sample $T_{current}$ from a base-10 log grid of 10 points and range $[T_{\min}, T_{\max}]$, including endpoints; or (ii) use per-sample adaptation (marked 'full seq.' in Table B7 and Table B8) and HAG run over the training sample with $T_{current} = L_{\text{seq}}$, then proceed to the next.
- *mean-HAG:*
  - **Rate Spread (β_r):** Spread parameter controlling the activity deviation from target ρ_r.
  - **Rate Target (ρ_r):** Target mean activity of neuron states.
- *variance-HAG:*
  - **Variance Target (ρ_v):** Target variance activity of neuron states.
  - **Variance Spread (β_v):** Spread parameter controlling sensitivity to deviations from target variance ρ_v.
  - **Saturation Threshold (θsat):** Threshold beyond which intrinsic plasticity mechanisms reduce synaptic weights.
  - **Saturation Scaling ($\eta_{\mathbf{sat}}$):** Factor by which synaptic weights are scaled when saturation occurs.

C. LSTM and GRU hyper-parameters

- **Hidden size** (n) – dimensionality of the RNNs's hidden state vector.
- **Number of layers** (N_{layers}) – number of stacked RNN layers, always one to have meaningful comparison with our other models.
- **Drop-out** (p_{drop}) – probability of zeroing elements of the hidden state before the final output layer, used for regularization.
- **Bidirectional flag** (b) – whether to use a bidirectional RNN (processing input in both forward and backward directions).
- **Learning rate** (η) – step size for the Adam optimizer.
- **Batch size** (B) – number of sequences processed per optimization step.

- **Epochs** (E) – number of passes over the entire labeled training dataset.

B.2 Hyperparameter Optimization Framework

The results in Figure B1 demonstrate that TPE achieves comparable or superior performance relative to CMA-ES (Hansen and Ostermeier, 2001), justifying its selection as the preferred optimization method. In particular, TPE demonstrates higher average scores and lower variability, underscoring its robustness for hyperparameter tuning.

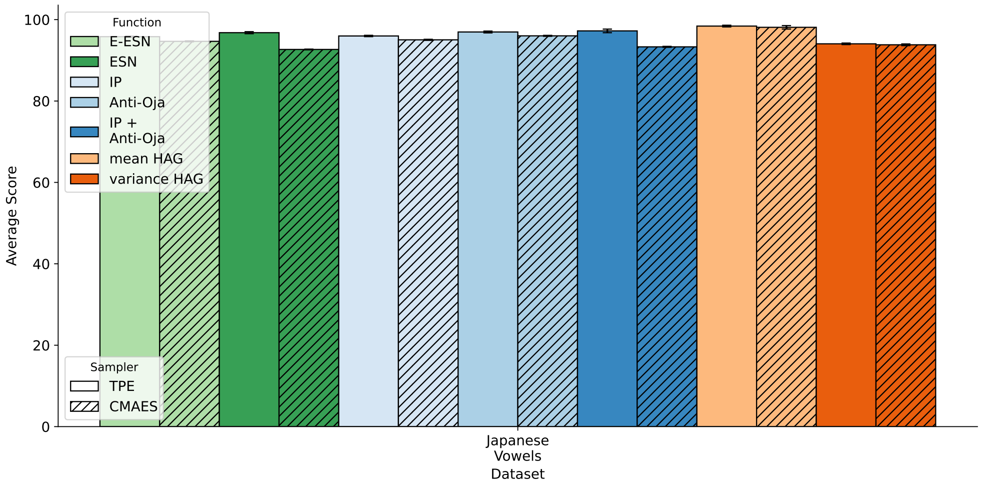

Fig. B1: Comparison of hyperparameter optimization performance between TPE and CMA-ES across different reservoir computing functions on the *Japanese Vowels* dataset. Error bars represent the standard deviation of the results.

B.3 Optimized Hyperparameters for Each Algorithm

Tables B3, B2, B4, B5, B6, B7 and B8 summarize the optimal hyperparameters found for each dataset and algorithm.

Table B2: Optimized hyperparameters for the E-ESN

Dataset	s_{in}	s_b	λ	p	ρ_s
**Japanese Vowels**	0.025	0.165	10^{-11}	0.06503	0.72
**CatsDogs**	0.105	0.005	10^{0}	0.20486	0.82
**FSDD**	0.105	0.005	10^{-4}	0.00472	0.91
**Spoken Arabic Digits**	0.010	0.040	10^{-11}	0.00353	1.01
**Speech Commands**	0.035	0.000	10^{-8}	0.36021	1.00
**Mackey-Glass**	0.195	0.170	10^{-12}	0.21140	0.40
**Lorenz**	0.155	0.080	10^{-9}	0.99326	0.50
**Sunspot daily**	0.095	0.080	10^{-12}	0.01406	1.38

Table B3: Optimized hyperparameters for the traditional ESN

Dataset	$\boldsymbol{s}_{\text{in}}$	$\boldsymbol{s_b}$	$\boldsymbol{\lambda}$	$\boldsymbol{p}$	$\boldsymbol{\rho_s}$
**Japanese Vowels**	0.150	0.050	10^{-1}	0.31821	0.85
**CatsDogs**	0.030	0.175	10^{-1}	0.74917	0.62
**FSDD**	0.08	0.035	10^{-4}	0.31952	0.98
**Spoken Arabic Digits**	0.025	0.065	10^{-9}	0.72228	1.03
**Speech Commands**	0.190	0.145	10^{-5}	0.97954	1.19
**Mackey-Glass**	0.185	0.145	10^{-10}	0.92745	0.40
**Lorenz**	0.115	0.090	10^{-3}	0.91559	0.94
**Sunspot daily**	0.010	0.025	10^{-12}	0.26144	0.55

Table B4: Optimized hyperparameters for IP

Dataset	$\boldsymbol{s}_{\text{in}}$	$\boldsymbol{s_b}$	$\boldsymbol{\lambda}$	$\boldsymbol{p}$	$\boldsymbol{\rho_s}$	$\boldsymbol{\mu}$	$\boldsymbol{\sigma}_{\text{ip}}$
**Japanese Vowels**	0.025	0.120	10^{-3}	0.82061	0.89	0.20637	0.66652
**CatsDogs**	0.185	0.105	10^{1}	0.10605	0.93	0.31114	0.11898
**FSDD**	0.185	0.105	10^{0}	0.90119	0.89	0.25828	0.18194
**Spoken Arabic Digits**	0.025	0.200	10^{-8}	0.22223	1.39	0.00061	0.14368
**Speech Commands**	0.015	0.070	10^{-3}	0.70052	1.60	0.33030	0.17147
**Mackey-Glass**	0.155	0.065	10^{-11}	0.80864	0.40	0.98106	0.39367
**Lorenz**	0.145	0.145	10^{-2}	0.64031	0.99	0.18872	0.12845
**Sunspot daily**	0.025	0.035	10^{-12}	0.87499	0.44	0.03698	0.70829

Table B5: Optimized hyperparameters for Anti-Oja

Dataset	s_{in}	s_b	λ	p	ρ_s	η_{oja}
**Japanese Vowels**	0.070	0.100	10^{-2}	0.11958	0.92	1.4×10^{-8}
**CatsDogs**	0.165	0.050	10^{1}	0.38232	0.75	8.2×10^{-8}
**FSDD**	0.025	0.06	10^{-7}	0.14171	0.98	1.3×10^{-5}
**Spoken Arabic Digits**	0.025	0.015	10^{-11}	0.59750	1.02	3.0×10^{-6}
**Speech Commands**	0.020	0.180	10^{-2}	0.54165	1.13	3.6×10^{-8}
**Mackey-Glass**	0.180	0.175	10^{-11}	0.84613	0.40	5.1×10^{-7}
**Lorenz**	0.185	0.040	10^{-5}	0.40514	0.73	3.1×10^{-7}
**Sunspot daily**	0.030	0.035	10^{-12}	0.64653	0.53	1.9×10^{-4}

Table B6: Optimized hyperparameters for IP + Anti-Oja

Dataset	s_{in}	s_b	λ	p	ρ_s	μ	σ	η_{lr}	η_{oja}
**Japanese Vowels**	0.145	0.045	10^{-1}	0.8244	0.68	0.108	0.692	1.15×10^{-4}	4.03×10^{-7}
**CatsDogs**	0.105	0.200	10^{0}	0.5177	1.06	0.578	0.0476	3.21×10^{-6}	3.27×10^{-8}
**FSDD**	0.155	0.065	10^{-3}	0.8343	0.92	0.0529	0.926	1.89×10^{-6}	8.57×10^{-8}
**Spoken Arabic Digits**	0.140	0.050	10^{-7}	0.8151	1.60	1.62×10^{-4}	0.145	6.41×10^{-4}	2.76×10^{-8}
**Speech Commands**	0.015	0.200	10^{-7}	0.5821	1.31	0.413	0.082	1.50×10^{-5}	3.55×10^{-8}
**Mackey-Glass**	0.195	0.145	10^{-12}	0.7799	0.44	0.254	0.159	2.73×10^{-6}	6.63×10^{-7}
**Lorenz**	0.125	0.195	10^{-3}	0.6329	0.96	0.423	0.618	1.82×10^{-6}	3.73×10^{-6}
**Sunspot daily**	0.010	0.130	10^{-12}	0.7448	0.43	0.366	0.918	1.24×10^{-6}	4.51×10^{-5}

Table B7: Optimized hyperparameters for the mean-HAG algorithm

Dataset	$\boldsymbol{s}_{\text{in}}$	$\boldsymbol{s_b}$	$\boldsymbol{\lambda}$	$\boldsymbol{\rho_r}$	$\boldsymbol{\beta_r}$	$\boldsymbol{\delta w}$	$\boldsymbol{T}_{\text{min}}$	$\boldsymbol{T}_{\text{max}}$
**Japanese Vowels**	0.14	0.14	10^{-4}	0.76	0.38	0.044	*full seq.*	*full seq.*
**CatsDogs**	0.010	0.005	10^{1}	0.52	0.365	0.022	*full seq.*	*full seq.*
**FSDD**	0.010	0.045	10^{-6}	0.51	0.39	0.015	309	414
**Spoken Arabic Digits**	0.015	0.015	10^{-11}	0.55	0.37	0.018	8	18
**Speech Commands**	0.010	0.000	10^{-12}	0.54	0.39	0.007	272	1477
**Mackey-Glass**	0.195	0.100	10^{-12}	0.50	0.35	0.099	153	1417
**Lorenz**	0.190	0.010	10^{-10}	0.77	0.02	0.011	139	1717
**Sunspot daily**	0.190	0.170	10^{-10}	0.82	0.32	0.094	441	1001

Table B8: Optimized hyperparameters for the variance-HAG algorithm

Dataset	s_{in}	s_b	λ	ρ_v	β_v	θ_{sat}	η_{sat}	δw	T_{min}	T_{max}
**Japanese Vowels**	0.190	0.040	10^{-3}	0.016	0.003	0.98	0.92	0.076	*full seq.*	*full seq.*
**CatsDogs**	0.100	0.035	10^{1}	0.014	0.007	0.86	0.92	0.065	*full seq.*	*full seq.*
**FSDD**	0.010	0.07	10^{-6}	0.013	0.005	0.82	0.98	0.05	*full seq.*	*full seq.*
**Spoken Arabic Digits**	0.015	0.025	10^{-11}	0.014	0.003	0.98	0.80	0.073	54	170
**Speech Commands**	0.010	0.000	10^{-9}	0.006	0.001	0.96	0.94	0.010	32	101
**Mackey-Glass**	0.085	0.185	10^{-12}	0.019	0.003	0.94	0.80	0.100	287	671
**Lorenz**	0.090	0.010	10^{-11}	0.018	0.001	0.84	0.86	0.007	53	1248
**Sunspot daily**	0.190	0.005	10^{-12}	0.017	0.005	0.86	0.84	0.046	252	1365

Table B9: Best LSTM hyperparameters for each dataset.

Dataset	n	p_{drop}	b	η	B	E
**Japanese Vowels**	384	0.16916	True	0.00434	16	19
**CatsDogs**	480	0.28738	True	1.13668e-4	8	7
**FSDD**	128	0.08223	False	0.00878	8	20
**Spoken Arabic Digits**	320	0.01123	False	0.00224	8	20
**Speech Commands**	448	0.42772	False	0.00166	32	20
**Mackey-Glass**	512	0.00065	False	0.00129	32	20
**Lorenz**	352	0.08908	True	0.00046	32	18
**Sunspot daily**	416	0.25248	False	0.00203	32	20

Table B10: Best GRU hyperparameters for each dataset.

**Dataset**	n	p_{drop}	$\mathbf{b}$	η	B	E
**Japanese Vowels**	160	0.38307	True	0.00305	8	20
**CatsDogs**	480	0.02875	False	1.79029e-04	8	18
**Spoken Arabic Digits**	224	0.18092	False	0.00185	8	19
**FSDD**	352	0.36602	True	0.0036	8	19
**Speech Commands**	512	0.46704	True	8.45589e-04	32	18
**Mackey–Glass**	224	1.72845e-04	False	4.08524e-04	8	20
**Lorenz**	256	0.296	False	1.00879e-04	8	19
**Sunspot daily**	320	0.07277	True	6.43770e-04	32	20

B.4 Cross validation performances

Table B11: Best cross-validation classification accuracy averaged over three folds. Bold values mark the highest accuracy for each data set; underlined values are second–best.

	Japanese Vowels	CatsDogs	FSDD	Spoken Arabic Digits	Speech Commands
**E-ESN**	96.67%	69.54%	29.5%	75.30%	5.84%
**ESN**	97.41%	70.73%	33.9%	89.20%	7.81%
**IP**	97.78%	**73.19%**	33.6%	90.68%	12.38%
**Anti-Oja**	97.78%	70.12%	34.6%	89.38%	9.37%
**IP + Anti-Oja**	97.78%	70.12%	33.9%	88.92%	11.82%
**LSTM**	90.00%	71.93%	59.6%	98.80%	85.63%
**GRU**	93.94%	73.15%	**74.6%**	**99.61%**	**89.43%**
**mean-HAG**	**99.26%**	71.35%	54.6%	97.68%	14.99%
**variance-HAG**	98.52%	72.56%	53.8%	98.05%	30.28%

Table B12: Best cross-validation NRMSE over 1 000 steps averaged over the three folds. Bold values are the lowest NRMSE (best); underlined values are the second–best.

	**Mackey-Glass**	**Lorenz**	**Sunspot daily**
**E-ESN**	0.00326	0.79343	0.45972
**ESN**	0.00453	0.77940	0.44683
**IP**	0.00437	0.77552	0.44667
**Anti-Oja**	0.00435	0.78661	0.44670
**IP + Anti-Oja**	0.00454	0.78154	0.44966
**LSTM**	0.03460	0.81612	0.21002
**GRU**	0.03429	**0.70520**	**0.20596**
**mean-HAG**	**0.00295**	0.77652	0.43092
**variance-HAG**	0.00491	0.78845	0.42448

After tuning, the winning configuration is re-trained on all available training folds (effectively 50 % more data) and then evaluated once on a disjoint test set. Two mechanisms therefore make a higher test score perfectly plausible (i) the benefit of more training data and (ii) the natural variance between folds and the fixed test split.

Conversely, whenever a test score drops below its CV counterpart (e.g. IP on Japanese Vowels, 97.8 % → 96.9 %) we attribute this to the usual validate–test gap: the chosen hyper-parameters slightly over-fitted the validation folds or the test set is distributionally harder. In all cases the gaps remain below ±2 percentage points, indicating a sound evaluation protocol.

Appendix C Additional Analysis

C.1 On Memory Capacity in a Multivariate Setting

Traditional metrics of linear memory capacity have proven effective for univariate ESNs, where the reservoir processes an independent random sequence and a linear readout can reconstruct delayed inputs. In our multivariate setting, however, the reservoir handles multiple, interdependent channels of data. This added complexity complicates the direct application of standard memory capacity tests, since each input channel may exhibit distinct temporal dynamics and inherent inter-channel correlations, making the notion of a single memory capacity measure ambiguous.

C.2 Computational Complexity

Baseline reservoir state update.

All reservoir variants (static ESN, excitatory ESN, IP, Anti-Oja, HAG) share their update equation:

$$\mathbf{x}_{t+1} = \sigma\big(W\mathbf{x}_t + W_{\text{in}}\mathbf{u}_t + \mathbf{b}\big),$$

costing per time step

$$\sim pn^2 + nd,$$

(sparse/dense recurrent multiplication plus input projection and elementwise nonlinearity). Over L_{pre} unsupervised steps:

$$\boxed{\sim L_{pre}(pn^2 + nd).}$$

Intrinsic Plasticity

Each neuron i maintains a gain a_i and bias b_i, updated online via stochastic gradient descent to match a target Gaussian firing distribution $\mathcal{N}(\mu, \sigma^2)$. Concretely, for the scalar activation

$$y_i = \tanh(a_i x_i + b_i),$$

the updates are (see Schrauwen et al (2008)):

$$\Delta b_i = -\eta \left(-\frac{\mu}{\sigma^2} + \frac{y_i}{\sigma^2} \left(2\sigma^2 + 1 - y_i^2 + \mu y_i\right) \right), \quad \Delta a_i = \frac{\eta}{a_i} + \Delta b_i x_i.$$

Each of these two updates requires only a constant number of element-wise multiplies, adds, and divides—on the order of 10 arithmetic operations per neuron. Hence the per-step cost is

$$\sim 10\, n,$$

and over a pretraining stream of length L_{pre} it sums to

$$\boxed{\sim 10\, L_{pre}\, n,}$$

which remains lower order compared to the reservoir's $\sim pL_{pre}\, n^2$ recurrent-multiply cost

Anti-Oja's rule.

At each time step t, every existing synapse w_{ij} is updated according to the "anti-Oja" rule, which actively decorrelates neuron activities by driving weights away from co-active pairs. The canonical update is

$$\Delta w_{ij}(t) \;=\; \eta\, y_j(t)\, \big[x_i(t) \;-\; y_j(t)\, w_{ij}(t)\big],$$

where $x_i(t)$ is the pre-synaptic activity, $y_j(t)$ is the post-synaptic activation of neuron j, and η is the learning rate. No further normalization is required, since the "anti-Oja" term inherently balances growth and decay.

- *Per-synapse cost:* compute $y_j x_i$ (1 multiply), compute $y_j^2 w_{ij}$ (2 multiplies), subtract (1 add), scale by η (1 multiply) $\Rightarrow \approx 5$ flops per active synapse.

With approximately $p\, n^2$ nonzero weights, the dominant per-timestep complexity is

$$\sim 5\, p\, n^2,$$

and over a pretraining stream of length L_{pre} this accumulates to

$$\boxed{\sim 5\, L_{pre}\, p\, n^2.}$$

Event-driven HAG overhead.

HAG does *not* update all synapses every step. After each adaptation window of length T_e it:

1. Accumulates activity statistics for n neurons during the window (incrementally): $\sim nT_e$.
2. Computes the Δz for the n neurons $\sim 2n$
3. Computes all pairwise Pearson correlations (Equation A1 inside the active set: $\sim s_e^2 T_e$.
4. Applies at most one synapse growth/pruning per active neuron: $\sim s_e$.

Event cost: $\sim nT_e + s_e^2 T_e$. Summing over K events gives the HAG overhead

$$\sim \sum_{e=1}^{K} (n + s_e^2) T_e.$$

At worst case $s_e = n$ for all e (empirically $s_e \ll n$ after early adaptation):

$$\sim n^2 \sum_e T_e \text{ (by keeping only the dominant term)}$$

which gives, since time-windows are non-overlapping, $\sum_e T_e = L_{pre}$,

$$\boxed{\sim n^2 L_{pre}}.$$

Readout training (ridge regression).

The linear readout training involves two distinct phases:

1. *State Collection*: computing and storing reservoir states for all N_{seq} labelled sequences (mean length $\bar{L}$), incurring a complexity of:

$$\sim N_{seq}\bar{L}(pn^2 + nd).$$

2. *Linear Regression Solving*: forming the covariance matrices ($X^\top X$ and $X^\top Y$) from collected dense states, costing:

$$\sim N_{seq}\bar{L}n^2,$$

and solving the ridge regression problem $(X^\top X + \lambda I)W = X^\top Y$ via Cholesky decomposition, adding:

$$\sim n^3 \quad (\text{or } \sim n^2 n_{\text{out}} \text{ for multiple outputs}).$$

Thus, the total complexity of readout training is:

$$\boxed{\sim N_{seq}\bar{L}(pn^2 + nd) + N_{seq}\bar{L}n^2 + n^3}.$$

LSTM/GRU + BPTT + Adam.

A single-layer LSTM contains $g = 4$ gate affine blocks (W_x, W_h, b) and GRU contains $g = 3$; the bias terms contribute only $\sim n)$ additions and are therefore negligible next to the two matrix–vector multiplies. The total number of trainable parameters is

$$P = n^2 + nd + n.$$

Forward pass – sequence level. For a sequence of length L we:

(a) **Gate affines** at every step

$$\sim g\,(nd + n^2) \quad \text{with } g = 4 \text{ or } 3.$$

(b) **Element-wise gate nonlinearities & cell updates** $\sim g\,n$ per step (absorbed by the previous term).

(c) **Store activations and cell states.** No arithmetic, but $\sim LP$ memory traffic so that the whole sequence can be unrolled later during back-propagation-through-time (BPTT).

Backward pass – one loss at $t = L - 1$. Although we compute the *loss only once, after the full sequence is seen*, its gradient must be propagated through all L time steps. The cost of the backward affine multiplications therefore mirrors the forward ones:

$$\sim g\,(nd + n^2) \quad \text{per step.}$$

The usual per-step output-layer operations that appear in sequence-to-sequence training vanish here (only one loss), so the constant in front of the asymptotic expression is slightly smaller than in the fully unrolled case.

Parameter update (Adam). After gradients have been accumulated we perform the Adam update once per step:

$$\sim P = n^2 + nd.$$

Putting it together. A *single* training time step (forward storage + backward + Adam) therefore costs

$$\sim \big(g\,(n^2+nd)\big)_{\text{fwd}} + \big(g\,(n^2+nd)\big)_{\text{bwd}} + \big(n^2+nd\big)_{\text{Adam}} =\sim (2g+1)\,(n^2+nd).$$

For N_{seq} sequences of *average* length $\bar{L}$ and E optimisation epochs the overall complexity is

$$\boxed{\sim (2g+1)\,E\,N_{\text{seq}}\,\bar{L}\,(n^2+nd)}$$

C.3 Estimation of computation costs

Representative concrete operation counts (Table C13) confirm: HAG's adaptive overhead is comparable to readout training and dramatically lower than multi-epoch gradient training, while delivering richer representations and higher accuracy than static or purely locally plastic reservoirs. In short, HAG yields a favorable accuracy–efficiency trade-off—approaching the performance of fully trained recurrent models at a fraction of their computational cost.

Table C13: Approximate floating–point operations (flops) for representative settings ($n = 500$, $d = 20$, $p = 0.1$, $N_{\text{seq}}\bar{L} = 10^5$, $E = 20$, $\overline{T} = 10$).

**Method**	Forward ($\times 10^9$)	Adaptation ($\times 10^9$)	Readout / BPTT Train ($\times 10^9$)	Total ($\times 10^9$)	Total / Static ESN
Static ESN / E-ESN	26	–	25.1	51.1	1.00
IP	26	0.50	25.1	51.6	1.01
Anti-Oja / IP+Anti-Oja	26	12.5	25.1	63.6	1.24
HAG *(worst case)*	26	25	25.1	76.1	1.49
LSTM	–	–	4680	4680	91.6
GRU	–	–	3640	3640	71.2

Assumptions / derivations: Reservoir forward per step: $pn^2 + nd = 0.1 \cdot 250{,}000 + 10{,}000 = 260{,}000$ flops $\Rightarrow 26 \times 10^9$ over 10^5 steps. IP per step $\approx 10n = 5{,}000$ flops $\Rightarrow 5 \times 10^8$. Anti-Oja per step $\approx 5pn^2 = 125{,}000$ flops $\Rightarrow 1.25 \times 10^{10}$. HAG worst case $= 25 \times 10^9$. Readout: form Nn^2 (2.5×10^{10}) + n^3 (1.25×10^8) $\approx 2.51 \times 10^{10}$. LSTM per labeled step (forward+backward+Adam) $\approx 9(n^2 + nd) = 9 \cdot 260{,}000 = 2.34 \times 10^6$; total steps $EN_{\text{seq}}\bar{L} = 2 \times 10^6 \Rightarrow 4.68 \times 10^{12}$ flops. GRU per labeled step $\approx 7(n^2 + nd) = 7 \cdot 260{,}000 = 1.82 \times 10^6 \Rightarrow 3.64 \times 10^{12}$ flops.

C.4 Final connectivity details

This section provides an analysis of the connectivity matrices produced by the different algorithms. The visualizations in Figure C2 and Figure C3 reveal distinct patterns in the matrices depending on the dataset and the specific variant of the HAG algorithm used.

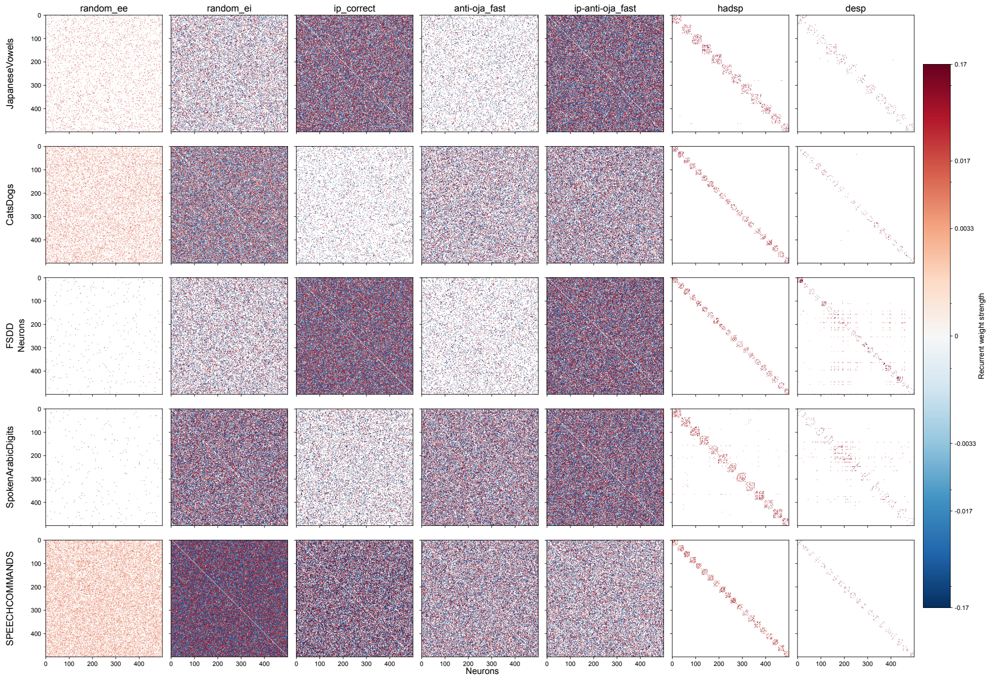

Fig. C2: Final connectivity matrices generated with the different methods across different classification datasets.

The connectivity matrices generated by HAG exhibit two notable characteristics. First, a modular patterns of strongly interconnected neurons. This structure corresponds to localized groups of neurons that represent specific features of the input space, reinforcing separability within subsets of the data. Second, there is a distinct set of connections corresponding to inter-cluster connectivity, which increases the overall decorrelation of the reservoir state by linking the most redundant subsets.

The matrices also reveal variance-HAG is the one that exhibits the most inter-cluster connectivity.

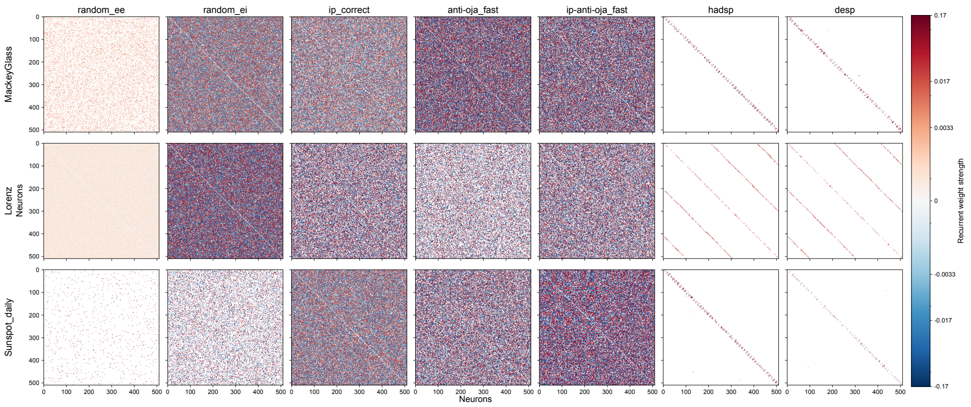

Fig. C3: Final connectivity matrices generated across different prediction datasets.

Appendix D Sensitivity to the Modularization of the Input Mapping

We compare two input–to–reservoir mappings: (i) *modular*, where each input channel is routed to a dedicated block of k neurons (within–block duplication), and (ii) *random*, where the same channels are projected through a randomly sampled input matrix with identical sparsity and scale. We evaluate across the same reservoir variants as in the main study (E-ESN, ESN, IP, Anti-Oja, IP+Anti-Oja) and also include the mean-HAG and variance-HAG.

For each dataset–algorithm pair we compute the paired difference

$$\Delta_{\text{pp}} = \text{Accuracy}_{\text{modular}} - \text{Accuracy}_{\text{random}} \quad \text{(percentage points, pp)},$$

so that $\Delta_{\text{pp}} > 0$ favors modularization. To isolate the effect of mapping, both conditions share reservoir size, sparsity, input scaling, and spectral–radius ranges. Hyperparameters are tuned *separately* for each mapping. To probe whether tuning difficulty could bias results, we also performed "cross–evaluations" that fix the best hyperparameters from one mapping and re–run the other; the direction of the pooled effect was preserved.

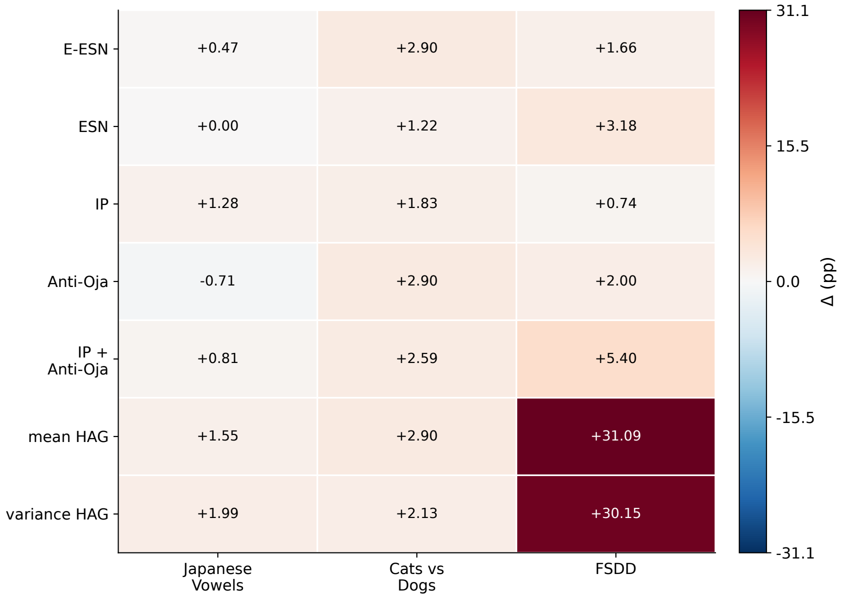

Fig. D4: **Sensitivity to input modularization.** Heatmap of Δ_{pp} (Modular − Random) across datasets and learning rules. Warm colors indicate a modularization benefit; cool colors indicate a benefit for a random input mapping. Values are means over seeds; standard deviations were modest and are omitted for readability.

Main findings.

Across 21 paired comparisons (3 datasets $\times$ 7 rules), the average improvement of modular over random input mapping is

$$\overline{\Delta_{\mathrm{pp}}} \;=\; 4.58 \text{ pp.}$$

Signs break 19 positive, 1 negative, and 1 tie. Gains are consistent on *Cats vs Dogs* (all rules +1.22 to +2.90 pp) and *Japanese Vowels* (mostly small positives; Anti-Oja is the lone negative at −0.71 pp; ESN ties at 0.00 pp). The largest effects occur on *FSDD*, where all rules are positive and the HAG variants are particularly strong (mean-HAG +31.09 pp; variance-HAG +30.15 pp) due to HAG completely breaking with the random input projection.

Appendix E Detailed Analysis Results

Table E14 and E15 summarize the spectral radius, average linear correlation, and CEVD for each combination of dataset and reservoir configuration. The spectral radius is measured from the connectivity matrix of the different networks obtained with various rules, while linear correlation and CEVD are measured based on neurons' activity during the test set inference for each dataset.

Table E14: Spectral radius (SR), Linear correlation (Corr), cumulative explained–variance dimension (CEVD) and distance correlation (dCor) (mean ± s.d. over four trials) on the prediction benchmarks.

Dataset	Alg.	SR	Corr.	CEVD	dCor
**Mackey-Glass**	E-ESN	0.40±0.00	0.753±0.008	3.5±0.5	0.741±0.004
	ESN	0.40±0.00	0.453±0.007	7.0±0.0	0.479±0.003
	IP	0.40±0.01	0.465±0.010	7.0±0.0	0.485±0.009
	Anti-Oja	0.40±0.01	0.455±0.008	7.0±0.0	0.478±0.007
	IP + Anti-Oja	0.44±0.00	0.442±0.010	7.8±0.4	0.453±0.005
	mean-HAG	0.95±0.01	0.509±0.002	6.0±0.0	0.533±0.001
	variance-HAG	1.89±0.03	0.399±0.011	6.3±0.4	0.486±0.007
**Lorenz**	E-ESN	0.50±0.00	0.833±0.001	3.0±0.0	0.800±0.002
	ESN	0.94±0.01	0.355±0.011	9.8±0.4	0.365±0.024
	IP	1.00±0.01	0.333±0.017	10.0±0.7	0.326±0.011
	Anti-Oja	0.73±0.00	0.400±0.015	8.0±0.0	0.403±0.008
	IP + Anti-Oja	0.97±0.02	0.479±0.301	8.5±3.2	0.381±0.100
	mean-HAG	0.21±0.02	0.564±0.000	6.0±0.0	0.531±0.000
	variance-HAG	0.21±0.02	0.564±0.000	6.0±0.0	0.532±0.000
**Sunspot (daily)**	E-ESN	1.38±0.00	0.959±0.002	1.0±0.0	0.846±0.002
	ESN	0.55±0.01	0.384±0.021	12.8±0.4	0.446±0.010
	IP	0.45±0.01	0.390±0.007	12.0±0.0	0.436±0.009
	Anti-Oja	0.52±0.01	0.339±0.003	13.0±0.0	0.391±0.003
	IP + Anti-Oja	0.43±0.01	0.327±0.005	13.0±0.0	0.377±0.014
	mean-HAG	1.12±0.00	0.728±0.010	6.8±0.4	0.530±0.002
	variance-HAG	1.54±0.02	0.494±0.010	13.8±0.4	0.530±0.010

Table E15: Spectral radius (SR), Linear correlation (Corr), cumulative explained–variance dimension (CEVD) and distance correlation (dCor) (mean ± s.d. over four trials) on the classification benchmarks.

Dataset	Alg.	SR	Corr	CEVD	dCor
**Japanese Vowels**	E-ESN	0.72±0.00	0.472±0.009	7.0±0.0	0.380±0.006
	ESN	0.85±0.01	0.256±0.005	17.8±0.8	0.251±0.005
	IP	0.89±0.01	0.241±0.007	22.3±1.1	0.241±0.009
	Anti-Oja	0.91±0.01	0.250±0.014	21.0±0.7	0.254±0.016
	IP + Anti-Oja	0.69±0.01	0.260±0.032	22.8±3.0	0.246±0.022
	mean-HAG	0.98±0.02	0.277±0.003	8.5±0.5	0.308±0.001
	variance-HAG	1.24±0.11	0.239±0.003	9.0±0.0	0.309±0.003
**CatsDogs**	E-ESN	0.82±0.00	0.755±0.009	7.3±0.4	0.687±0.008
	ESN	0.61±0.01	0.181±0.008	33.0±0.7	0.182±0.007
	IP	0.93±0.01	0.197±0.023	68.5±1.1	0.193±0.008
	Anti-Oja	0.77±0.02	0.187±0.007	43.3±2.2	0.191±0.007
	IP + Anti-Oja	1.07±0.02	0.185±0.003	38.5±1.1	0.186±0.006
	mean-HAG	1.01±0.00	0.465±0.011	13.0±0.0	0.291±0.000
	variance-HAG	1.67±0.02	0.207±0.008	10.8±0.4	0.289±0.006
**FSDD**	E-ESN	0.97±0.00	0.956±0.001	1.5±0.5	0.856±0.011
	ESN	1.01±0.01	0.466±0.171	17.3±25.3	0.444±0.199
	IP	1.27±0.01	0.182±0.036	37.5±4.4	0.156±0.018
	Anti-Oja	1.17±0.01	0.095±0.005	67.0±4.7	0.090±0.003
	IP + Anti-Oja	1.39±0.03	0.221±0.074	66.3±4.3	0.193±0.039
	mean-HAG	1.01±0.00	0.423±0.006	12.0±0.0	0.258±0.000
	variance-HAG	2.89±0.00	0.313±0.005	14.8±0.4	0.224±0.003
**Spoken Arabic Digits**	E-ESN	1.01±0.00	0.439±0.006	10.3±0.4	0.450±0.016
	ESN	1.03±0.01	0.616±0.128	7.0±8.7	0.549±0.159
	IP	1.38±0.01	0.414±0.187	12.8±14.2	0.386±0.161
	Anti-Oja	1.01±0.02	0.394±0.117	14.3±11.8	0.371±0.125
	IP + Anti-Oja	1.60±0.02	0.293±0.090	24.3±8.0	0.363±0.202
	mean-HAG	1.00±0.00	0.496±0.021	9.0±0.0	0.310±0.000
	variance-HAG	2.38±0.01	0.279±0.004	10.3±0.4	0.316±0.001
**Speech Commands**	E-ESN	1.00±0.00	0.994±0.000	1.0±0.0	0.993±0.000
	ESN	1.19±0.01	0.386±0.051	22.5±18.4	0.401±0.168
	IP	1.61±0.04	0.256±0.070	28.3±13.8	0.238±0.115
	Anti-Oja	1.13±0.01	0.277±0.088	18.0±6.2	0.291±0.118
	IP + Anti-Oja	1.22±0.01	0.346±0.084	9.8±4.4	0.416±0.124
	mean-HAG	1.01±0.00	0.613±0.003	9.0±0.0	0.661±0.001
	variance-HAG	1.98±0.03	0.509±0.007	12.3±0.4	0.587±0.007

Table E16: Class–separability and clustering scores (mean ± s.d. over four trials). SepR = separability ratio, Silh = silhouette coefficient, DB = Davies–Bouldin index, CH = Calinski–Harabasz index.

Dataset	Alg.	SepR	Silh	DB	CH
**Japanese Vowels**	E-ESN	1.51±0.02	0.257±0.003	1.37±0.02	87.92±3.16
	ESN	1.26±0.03	0.226±0.016	1.49±0.07	61.67±3.63
	IP	1.22±0.07	0.214±0.027	1.55±0.09	58.55±7.20
	Anti-Oja	1.19±0.05	0.178±0.027	1.69±0.16	54.32±4.80
	IP + Anti-Oja	1.11±0.05	0.174±0.009	1.75±0.06	46.60±5.52
	mean-HAG	1.46±0.01	0.278±0.004	1.25±0.01	79.41±1.28
	variance-HAG	1.58±0.01	0.317±0.002	1.25±0.01	91.66±2.77
**CatsDogs**	E-ESN	0.15±0.00	0.016±0.001	9.06±0.18	1.32±0.05
	ESN	0.25±0.01	0.026±0.003	5.68±0.32	4.78±0.57
	IP	0.24±0.06	0.024±0.010	6.24±1.29	4.49±2.37
	Anti-Oja	0.25±0.03	0.026±0.005	5.57±0.54	5.08±1.08
	IP + Anti-Oja	0.25±0.01	0.027±0.002	5.54±0.31	5.01±0.53
	mean-HAG	0.33±0.01	0.047±0.004	4.20±0.18	8.11±0.72
	variance-HAG	0.32±0.01	0.043±0.002	4.38±0.14	7.43±0.48
**FSDD**	E-ESN	0.35±0.03	0.173±0.003	−0.07±0.01	15.99±1.51
	ESN	0.56±0.08	0.208±0.017	−0.03±0.00	10.55±0.53
	IP	2.67±1.36	0.215±0.054	−0.03±0.01	11.27±2.05
	Anti-Oja	0.22±0.05	0.218±0.026	−0.03±0.01	10.30±0.67
	IP + Anti-Oja	1.44±0.43	0.221±0.012	−0.03±0.01	9.72±0.37
	mean-HAG	0.14±0.01	0.428±0.009	0.00±0.00	6.44±0.12
	variance-HAG	0.09±0.01	0.379±0.023	−0.01±0.00	7.25±0.54
**Spoken Arabic Digits**	E-ESN	0.56±0.03	−0.054±0.003	9.02±0.77	78.60±7.92
	ESN	0.18±0.18	−0.046±0.025	51.92±34.06	12.56±19.72
	IP	0.09±0.02	−0.048±0.013	37.90±6.14	1.39±0.41
	Anti-Oja	0.16±0.08	−0.036±0.013	18.88±7.81	7.19±7.51
	IP + Anti-Oja	0.36±0.15	−0.030±0.015	8.60±2.82	34.11±27.20
	mean-HAG	0.65±0.01	0.007±0.001	5.14±0.07	90.28±2.45
	variance-HAG	0.61±0.01	0.006±0.001	5.41±0.10	82.25±3.07
**Speech Commands**	E-ESN	0.26±0.00	−0.207±0.001	157.94±8.25	15.64±0.15
	ESN	0.07±0.05	−0.035±0.033	44.03±15.01	1.90±2.28
	IP	0.05±0.00	−0.015±0.002	36.16±3.22	0.84±0.14
	Anti-Oja	0.06 ± 0.02	−0.019 ± 0.007	54.55 ± 27.12	1.03 ± 0.79
	IP + Anti-Oja	0.06 ± 0.03	−0.039 ± 0.014	147.50 ± 160.49	1.24 ± 1.28
	mean-HAG	0.24±0.00	−0.104±0.001	19.40±0.15	15.45±0.17
	variance-HAG	0.27±0.00	−0.099±0.002	16.45±0.32	19.99±0.17

References

Abreu S, Boikov I, Goldmann M, et al (2024) A photonics perspective on computing with physical substrates. Reviews in Physics 12:100093. https://doi.org/10.1016/j.revip.2024.100093

Hansen N, Ostermeier A (2001) Completely derandomized self-adaptation in evolution strategies. Evolutionary Computation 9(2):159–195. https://doi.org/10.1162/106365601750190398

Kudithipudi D, Saleh Q, Merkel C, et al (2016) Design and analysis of a neuromemristive reservoir computing architecture for biosignal processing. Frontiers in Neuroscience 9. https://doi.org/10.3389/fnins.2015.00502

Pearson K (1895) Vii. note on regression and inheritance in the case of two parents. Proceedings of the Royal Society of London 58(347–352):240–242. https://doi.org/10.1098/rspl.1895.0041

Schrauwen B, Wardermann M, Verstraeten D, et al (2008) Improving reservoirs using intrinsic plasticity. Neurocomputing 71(7–9):1159–1171. https://doi.org/10.1016/j.neucom.2007.12.020

Wang D, Nie Y, Hu G, et al (2024) Ultrafast silicon photonic reservoir computing engine delivering over 200 tops. Nature Communications 15(1). https://doi.org/10.1038/s41467-024-55172-3
